# 

**Published:** 1894-05

**Authors:** 


### CONTENTS.

### COMMUNICATIONS.

Infection from the Mouth .......... 209
Devitalization of Dental Pulp .......... 218
Diseases of the Maxillary Bones and their Periosteum .......... 223

### COMMENCEMENTS.

American College of Dental Surgery .......... 230
Royal College of Dental Surgeons of Ontario .......... 230
Ohio College of Dental Surgery .......... 231
Indiana Dental College .......... 232
Baltimore College of Dental Surgery .......... 232
New York College of Dentistry .......... 233
Philadelphia Dental College .......... 233
University of Maryland .......... 234
Cincinnati College of Dental Surgery .......... 235
Meharry Medical College .......... 235
Kansas City Dental College .......... 236
Ohio Medical University .......... 236
Western Reserve University .......... 236
University of Iowa .......... 237
The Cleveland University of Medicine and Surgery .......... 237
Pennsylvania College of Dental Surgery .......... 238
Atlanta Dental College .......... 239
Missouri Dental College .......... 239
Western Dental College .......... 240
University of Denver .......... 240
Vanderbilt University .......... 241
Illness Ascribed to Dentition .......... 241

### SELECTIONS.

Root Filling .......... 242

### NOTICES.

Programme for Dental Section of American Medical Association .......... 250
Chicago Dental Society .......... 251
The Midwinter Fair, San Francisco .......... 251
The Dental Society of the State of New York .......... 252
The Texas State Dental Association .......... 253
CORRESPONDENCE .......... 253

### EDITORIAL.

Bibliographical .......... 254
Necrology .......... 255

